# Simulation-Based Training in Emergency Obstetric Care in Sub-Saharan and Central Africa: A Scoping Review

**DOI:** 10.5334/aogh.3891

**Published:** 2023-09-28

**Authors:** Anne A. C. van Tetering, Peter Ntuyo, Renata P. J. Martens, Naomi Winter, Josaphat Byamugisha, S. Guid Oei, Annemarie F. Fransen, M. Beatrijs van der Hout-van der Jagt

**Affiliations:** 1 Department of Obstetrics and Gynaecology, Máxima Medical Center, Veldhoven, NL; 2 Department of Obstetrics and Gynaecology, Amphia Hospital, Breda, NL; 3 Department of Obstetrics and Gynaecology, Mulago Specialised Women and Neonatal Hospital, UG; 4 Department of Obstetrics and Gynaecology, St. Antonius Hospital, Utrecht, NL; 5 Department of Obstetrics and Gynaecology, Makerere University College of Health Sciences, UG; 6 Department of Electrical Engineering, Eindhoven University of Technology, Eindhoven, NL; 7 Department of Biomedical Engineering Eindhoven University of Technology, Eindhoven, NL

**Keywords:** medical education, simulation training, obstetrics, instructional design, sub-Saharan and Central Africa

## Abstract

**Background:**

Every day approximately 810 women die from complications related to pregnancy and childbirth worldwide. Around two thirds of these deaths happen in sub-Saharan Africa. One of the strategies to decrease these numbers is improving the quality of care by emergency obstetric simulation-based training. The effectiveness of such training programs depends on the program’s instructional design.

**Objective:**

This review gives an overview of studies about emergency obstetric simulation-based training and examines the applied instructional design of the training programs in sub-Saharan and Central Africa.

**Methods:**

We searched Medline, Embase and Cochrane Library from inception to May 2021. Peer-reviewed articles on emergency obstetric, postgraduate, simulation-based training in sub-Saharan and Central Africa were included. Outcome measures were categorized based on Kirkpatrick’s levels of training evaluation. The instructional design was evaluated by using the ID-SIM questionnaire.

**Findings:**

In total, 47 studies met the inclusion criteria. Evaluation on Kirkpatrick level 1 showed positive reactions in 18 studies. Challenges and recommendations were considered. Results on knowledge, skills, and predictors for these results (Kirkpatrick level 2) were described in 29 studies. Retention as well as decay of knowledge and skills over time were presented. Results at Kirkpatrick level 3 were measured in 12 studies of which seven studies demonstrated improvements of skills on-the-job. Improvements of maternal and neonatal outcomes were described in fifteen studies and three studies reported on cost-estimations for training rollout (Kirkpatrick level 4). Instructional design items were heterogeneously applied and described.

**Conclusions:**

Results of 47 studies indicate evidence that simulation-based training in sub-Saharan and Central Africa can have a positive impact across all four levels of Kirkpatrick’s training evaluation model. However, results were not consistent across all studies and the effects vary over time. A detailed description of instructional design features in future publications on simulation-based training will contribute to a deeper understanding of the underlying mechanisms that determine why certain training programs are more effective in improving maternal and neonatal healthcare outcomes than other.

## Introduction

Despite an impressive worldwide drop in maternal mortality since 2000, every day approximately 810 women still die from preventable complications related to pregnancy and childbirth [1]. Roughly two-thirds of these deaths occur in sub-Saharan Africa [1]. The major complications responsible for these deaths are severe bleeding, infections, and high blood pressure during pregnancy, complications from delivery, and unsafe abortion [1]. Most of these complications are preventable or treatable, as the healthcare solutions to prevent or manage these situations are well known [1]. Factors that prevent women from receiving and seeking care for these situations are poverty, distance to health facilities, lack of knowledge, cultural beliefs and practices, but also inadequate healthcare services [2]. Barriers in these services include poor management of emergency obstetric care provision, delayed referral practices, and limited coordination among staff [1,2]. Simulation-based emergency obstetric training can be a valuable tool to enhance the performance of obstetric care teams.

The observation made by Black et al. in 2003 revealed a gap in the availability and evaluation of training programs in acute obstetric emergencies in both high-income countries and low- and middle income countries [3]. Since this observation, the number of obstetric simulation peer-reviewed reports has increased exponentially with merging evidence that simulation-based emergency obstetric training can improve healthcare provider knowledge and skills, clinical practice, and health outcomes [3,4,5,6,7,8,9,10]. However, these results were not consistent across all training programs. The prioritization of scaling up effective training packages was recommended with further evaluation research beyond the outcome-based Kirkpatrick levels to delve deeper into the mechanisms that drive or hinder the achievement of training outcomes [4,11].

Kirkpatrick’s theoretical model is a frequently used framework for evaluating the effectiveness of a training program [12]. This model contains four levels [12]. The first two levels assess trainees’ experience and learning in an educational setting, while level three and four shift to the effects on actual health workers’ behaviour and patient outcomes. The effectiveness of simulation-based training depends, among other things, on the instructional design of the training program. The instructional design is generally referred as the ‘set of prescriptions for teaching methods to improve the quality of instruction with a goal of optimizing learning outcomes’ [13]. The evidence from systematic reviews identified essential instructional design features for simulation-based medical education [14,15]. Evaluation of these features provides a deeper understanding of the strengths and weaknesses of training courses.

This review gives an overview of studies about emergency obstetric, postgraduate, simulation-based training in sub-Saharan and Central Africa, and provides insight into the attention given to the instructional design of training programs. The rationale for focusing on sub-Saharan and Central Africa was due to the persisting high maternal and neonatal mortality rates from preventable causes related to pregnancy and childbirth. Moreover, worldwide variations in ethnic and geographical perspectives, as well as local clinical settings, impact learning approaches and outcomes in educational settings [16].

## Materials and Methods

### Search strategy

We searched Medline, Embase and Cochrane Library from inception to May 2021. Keywords used for the search included combinations of ‘Obstetrics’ AND ‘Simulation training’ AND ‘Sub-Saharan and Central Africa’ (see Appendix 1 for the complete search strategy).

### Eligibility criteria

We selected all peer-reviewed articles on simulation-based training evaluation in obstetric emergencies including technical skills, non-technical skills or both, provided for obstetric qualified healthcare providers in sub-Saharan and Central Africa. We excluded editorials, opinions, conference abstract, study protocols, reviews, non-English publications, and articles describing courses for unqualified obstetric healthcare workers, including birth attendants without formal training.

Simulation training was defined as ‘an artificial representation of a real world process to achieve educational goals through experiential learning and is characterised by the use of simulation tools that serve as an alternative for real patients’ [17]. Additionally, articles were included when simulation-based training was applied as major component of obstetric quality improvement activities related directly to the direct causes of maternal and neonatal deaths. Obstetric emergencies were defined as complications that arise during pregnancy and childbirth that can threaten the well-being of mother and/or child [18]. Studies on obstetric training without simulation, and simulation-based training in medical fields other than obstetrics were excluded.

### Study selection

Two authors (AT and RM) independently reviewed all titles and abstracts. Based on title and abstract, full text articles were assessed for eligibility. Any disagreements were resolved by a third author (BH or AF).

### Data extraction and analysis

Data extraction was done independently by four authors (AT, RM, PT, NW). Any disagreements were resolved by discussion between the authors or, if required, by consultation of another author (BH). The characteristics of the included studies were extracted into a predesigned summary table and the strength of the evidence was appraised using the Oxford Centre for Evidence-Based Medicine (OCEBM, 2011) levels of evidence [19]. Outcome measures according to the four levels of Kirkpatrick’s model were summarized. To assess the instructional design of the training programs, each article was subjected to evaluation using the ID-SIM (Instructional Design of a Simulation Improved by Monitoring) questionnaire. The ID-SIM questionnaire is an evidence-based assessment tool comprising of 42 items. This tool serves a dual purpose, assisting both in the development and evaluation of a simulation-based team training [20]. The items represent ten instructional design features described by Issenberg et al. and McGaghie et al. Per instructional design features, the number of items ranges from two to six [14,15]. Examples of these instructional design features include feedback, repetitive practice, and ranging difficulty level. Rather than adopting the rating system validated within the ID-SIM questionnaire, we opted to quantify the addressed items from the questionnaire for each article. This decision was driven by the wide variation in the descriptions of instructional design items across the reviewed studies, which made a qualitative content-based evaluation impossible.

## Results

### Search results

**Figure 1:**
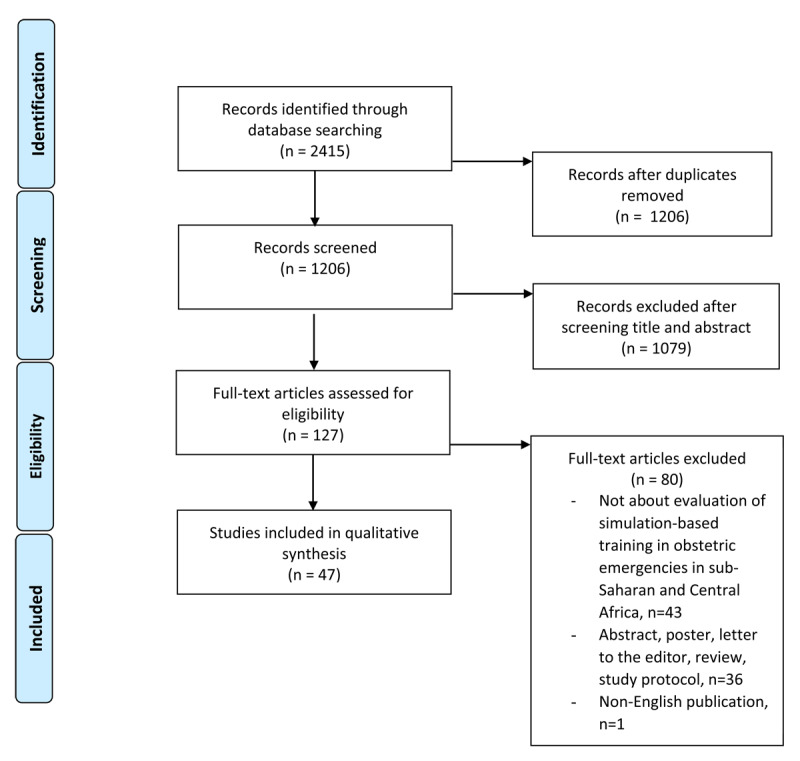
Study flow diagram to map the number of articles identified, included and excluded.

Details of the study selection process are depicted in Figure 1. From the identified 1206 unique records, 127 articles were selected according to the selection criteria after reading title and abstract. After examination of the 127 full articles, 80 articles were excluded. Among these, 43 articles were excluded as they did not report on simulation-based training in obstetric emergencies within the specified regions of sub-Saharan and Central Africa. Additionally, 36 articles were excluded due to their format, including abstracts, posters, letters to the editor, study protocols or reviews. Furthermore, one article was excluded for being non-English. Hence, a total of 47 peer-reviewed studies were included in this review.

### Study characteristics

Table 1 provides a detailed description of the study characteristics of the 47 included studies. The studies span a diverse array of study designs including eighteen pre-post studies [21,22,31,32,33,34,35,36,37,38,23,24,25,26,27,28,29,30], seven cluster-randomized controlled trials [39,40,41,42,43,44,45], five descriptive studies [46,47,48,49,50], two quasi-experimental studies [51,52], and one observational study [53]. Ten studies included both descriptive and pre-post data [54,55,56,57,58,59,60,61,62,63], and two studies included both descriptive and observational data [64,65]. In addition, two studies were cost analysis studies [66,67]. Five out of seven cluster-randomized controlled trials were published since 2018 [42,43,44,45,68].

**Table 1:** Characteristics of selected studies.


AUTHORS	YEAR	STUDY DESIGN	COMPARISON	COUNTRY	SETTING	NAME OF THE TRAINING PROGRAM	TRAINED POPULATION	UNI- OR INTERPROFESSIONAL	INTERVENTION	DURATION OF INTERVENTION	SCENARIO CONTENT	TECHNICAL SKILLS, NON-TECHNICAL SKILLS OR BOTH	QUALITY OF EVIDENCE (USING THE OXFORD CENTRE FOR EVIDENCE-BASED MEDICINE LEVELS OF EVIDENCE, 2011)	KIRKPATRICK’S LEVEL OF TRAINING EVALUATION	NUMBER OF DESCRIBED INSTRUCTIONAL DESIGN ITEMS (TOTAL OF 42 ID-SIM ITEMS)

**Afulani et al.**	2020	Descriptive and pre-post study	Pretraining vs. posttraining	Ghana	1 referral hospital, 4 health centers	None (based on PRONTO international curriculum)	Midwives, doctors, anesthetist, nurses	Interprofessional	Low-tech, highly realistic simulation and team training with facilitated debriefing	2 days with four 3-hour refresher training once a month	Normal birth, emergency obstetric and neonatal care, aspects of respectful maternity care	Both	2c	I, II	19

**Afulani et al.**	2019	Pre-post study	Pretraining vs. 6 months posttraining	Ghana	1 referral hospital, 4 health centers	None (based on PRONTO international curriculum)	Midwives, doctors, anesthetist, nurses	Interprofessional	Low-tech, highly realistic simulation and team training with facilitated debriefing	2 days with four 3-hour refresher training once a month	Normal birth, emergency obstetric and neonatal care, aspects of respectful maternity care	Both	2c	IV	20

**Alwy Al-Beity et al.**	2020	Pre-post study	Pretraining vs. posttraining vs. 10 months posttraining	Tanzania	23 district hospitals, 38 large health centres	Helping Mothers Survive: Bleeding After Birth	Medical doctors, other clinicians, nurse-midwives, auxiliary staff	Interprofessional	Facility-based simulation training using peer practioners and repetitive practice	1 day with weekly 30-40 minutes practice drills for 8 weeks	Basic delivery skills including active management of third stage of labour and management of PPH	Technical skills	2c	II	18

**Ameh et al.**	2016	Pre-post study	Pretraining vs. posttraining	Ghana, Kenya, Malawi, Nigeria, Sierra Leone, Tanzania, Zimbabwe, Bangladesh, Pakistan	Unknown	Emergency Obstetric and Newborn Care (EmOC&NC)	Doctors, medical officers, nurses, midwives, nursing aides	Interprofessional	Interactive skills and drills training using low fidelity simulators. Training includes lectures, workshops, role play, mentoring, and monitoring and evaluation	3 to 5 days	Major causes of maternal and newborn death and EmOC signal functions	Both	2c	II	6

**Ameh et al.**	2012	Descriptive and pre-post study	Pretraining vs. posttraining vs. 3 months posttraining vs. 6 months posttraining	Somaliland	1 public hospital, 2 private hospitals, 8 public health care clinics	Life Saving Skills – Emergency Obstetric and Newborn Care (LSS-EOC and NC)	Nurses, midwives, midwivery tutors, obstetricians, medical officers, medical interns, final-year medical and midwivery students	Interprofessional	Hands-on and context specific training using interactive simulation and didactic education	4 days	Direct causes of maternal death, EmOC signal functions, and competencies of skilled birth attendants	Both	2c	I, II, III, VI	7

**Andreatta et al.**	2011	Descriptive and pre-post study	Pretraining vs. posttraining, descriptive comments	Ghana	2 regional and 2 district medical centers	None	Nurse-midwives, nurse-midwifery students, traditional birth attendants	Uniprofessional	Hands-on and culturally specific training using a simulator Follow-up after instruction to encourage resiliency of the training effects	2 days	Postpartum haemorrhage	Technical skills	2c	I, II, III, IV	15

**Arabi et al.**	2016	Pre-post study	Pretraining vs. 3 months posttraining vs. 12 months posttraining	Sudan	–	Helping Babies Breathe	Village midwives	Uniprofessional	Hands-on practical training using a low-cost newborn simulator	Unknown	Basic newborn care and neonatal resuscitation	Technical skills	2c	II	14

**Arlington et al.**	2017	Descriptive and observational study	Posttraining vs. 4–6 week posttraining vs. 4–6 months posttraining	Tanzania	33 regional and district hospitals, 35 healthcenters, 163 dispensaries	Helping Babies Breathe	Medical doctors, assistant medical officers, clinical officers, assistant clinical officers, nurse or nurse-midwives, medical assistants, other health workers	Interprofessional	Hands-on practical training using a low-cost newborn simulator Followed by supportive supervision visits	1 day	Basic newborn care and neonatal resuscitation	Technical skills	2c	I, II	7

**Asiedu et al.**	2019	Descriptive study	None	Ghana	9 district and regional facilities	None	Obstetrician, medical officer, midwives, management, clinical supervision, pediatric nurse, general nurse	Interprofessional	Low-dose, high-frequency (LDHFT) in-service training coupled with mobile mentoring	2 4-day sessions and frequent practice during and after the training, weekly mobile mentoring during 1 year	Basic emergency obstetric and newborn care including newborn resuscitation, respectful maternity care and clinical decision-making	Both	5	I	18

**Bang et al.**	2016	Pre-post study	Pretraining vs. posttraining vs. 6 months posttraining	India, Kenya	health facilities that provided 24-h coverage for deliveries 7 days/week, with a minimum perinatal mortality rate of 30 per 1000 registry deliveries	Helping Babies Breathe	Providers from pediatrics, obstetrics, anesthesia, nursing departments, facility administrators	Interprofessional	Hands-on practical training using a low-cost newborn simulatorFollowed by ‘on-the-job’ and refresher training	3 days, half-day refresher course	Basic newborn care and neonatal resuscitation	Technical skills	2c	II	18

**Cavicchiolo et al.**	2018	Pre-post study	Pretraining vs. posttraining vs. after LDHF training	Mozambique	1 referral hospital	None	Midwives	Uniprofessional	Neonatal resuscitation program with 8 months later a LDHFT	Neonatal rescusitation program: duration unknownLDHFT: weekly 3-hour sessions for 6 months	Neonatal resuscitation	Non-technical skills	2c	II	18

**Chang et al.**	2019	Pre-post study	Pretraining vs. education period vs. posttraining	Malawi	1 tertiary referral hospital, 1 rural district health center	Alliance for Innovation on Maternal Health (AIM) Malawi program	Nurse midwives, clinicians, anesthetists, ancillary staff	Interprofessional	Classroom didactics, skills laboratory and simulation training	2 days	Prevention and management of postpartum haemorrhage, teamwork and communication	Both	2c	I, III, IV	15

**Chaudhury et al.**	2016	Cost-analysis study in a cross-sectional design	none	Tanzania	336 health facilities (dispensaries, health centers, hospitals)	Helping Babies Breathe	Health providers	Interprofessional	Hands-on practical training using a low-cost newborn simulator Followed by supportive supervision visits	1 day	Basic newborn care and neonatal resuscitation	Technical skills	2c	IV	16

**Dettinger et al.**	2018	Descriptive and pre-post study	Pretraining vs. Module 1 posttraining vs. Module 2 posttraining (3 months later)	Kenya	44 level 2 or 3 facilities, conducting 10 or more deliveries per year	PRONTO International simulation-based training	Medical officer, clinical officers, nurses	Interprofessional	Skills and drills training (the MoH Harmonized training package) Intervention facilities received additional PRONTO training covering a subset of the MoH Harmonized training package supplemented with team and simulation training	Both intervention and control group: 5 days (MoH Harmonized training package)Intervention group: additional 3 days (PRONTO training)	The MoH Harmonized training package: antenatal, intrapartum, and postnatal care PRONTO: obstetric heamorrhage, neonatal resuscitation, (Module 1), pre-eclampsia, shoulder dystocia, review of strategic goal achievement (Module 2), teamwork and communication (Module 1 and 2)	Both	2c	I, II, III	18

**Drake et al.**	2019	Quasi-experimental trial	2 training approaches, posttraining vs. 4–6 weeks posttraining	Tanzania	All public and faith-based health facilities across 16 of 26 mainland regions	Helping Babies Breathe	Nurses-midwives, medical attendants, other clinicians	Interprofessional	1. Initial training approach: hands-on practical training using a low-cost newborn simulator, followed by ‘on-the-job’ and supportive supervision visits2. Modified training approach: hands-on practical training using a low-cost newborn simulator followed by the use of a structured on-the-job training tool to facilitate self-learning as well as peer-to-peer continuous learning	1 day and possibility of self-initiated practice after the training day	Basic newborn care and neonatal resuscitation	Technical skills	2c	II	21

**Dumont et al.**	2013	Cluster-randomised controlled trial	Intervention vs. control group	Mali, Senegal	46 public first-level and second-level referral hospitals	Quality of care, Risk management and Technology in obstetrics (QUARITE)	Doctors, midwives, nurses	Interprofessional	Interactive workshop using the ALARM international course and outreach visits focused on maternal death reviews and best practice implementation	6 days workshop, quarterly educational outreach visits	Emergency obstetric care, topics were based on maternal death reviews. Most recurrent topics were pre-eclampsia and management of PPH	Both	1b	IV	13

**Eblovi et al.**	2017	Pre-post study	Posttraining vs. 4 months posttraining vs. 4 months after the refresher training	Ghana	Small rural health clinics	Helping Babies Breathe	Midwives	Uniprofessional	Hands-on practical training using a low-cost newborn simulatorFollowed by refresher training	2 days, 2 days refresher course after 1 year	Basic newborn care and neonatal resuscitation	Technical skills	2c	II, IV	14

**Egenberg et al.**	2017	Descriptive and exploratory study	None	Tanzania	1 consultant hospital and 1 regional referral hospital	Based on Helping Mothers Survive: Bleeding After Birth	Midwives, doctors, medical attendants	Interprofessional	Context-specific training based on the local protocol and HMS-BAB	Unknown	Basic delivery skills including active management of third stage of labour and management of PPH, communication	Both	5	I	12

**Ersdal et al.**	2013	Pre-post study	Pretraining vs. posttraining	Tanzania	1 rural referral hospital	Helping Babies Breathe	Midwives, anesthetic nurses, operating nurses, student nurses, ward attendants	Interprofessional	Hands-on practical training using a low-cost newborn simulator	1 day	Basic newborn care and neonatal resuscitation	Technical skills	2c	II, III	9

**Evans et al.**	2018	Pragmatic, cluster-randomised trial	Three training approaches Posttraining vs. 6 months posttraining vs. 12 months posttraining	Uganda	16 health centers level II, 76 health centers level III, 23 health centers level IV, 11 hospitals	None (based on Helping Babies Breathe and Helping Mothers Survive: Bleeding After Birth training modules)	All providers on the labor ward, not specified	Unknown	1. Facility-based, LDHF team training and ongoing practice2. As group 1 + peer-assisted learning component3. As group 2 + phone support	1 day HMS BAB with suggestion to practice for 10-15min once per week for 8 weeks, followed by 1 day HBB training, with suggestion to practicie 10-15min once per week for 8 weeks, followed by suggestion to practice both maternal and newborn scenarios for 4 weeks	Postpartum haemorrhage and neonatal resuscitation	Technical skills	1b	II, III, IV	12

**Evans et al.**	2014	Descriptive and observational study	Pretraining vs. posttraining	India, Malawi, Tanzania	Peripheral and higher-level public facilities	Helping Mothers Survive: Bleeding After Birth	Health orderlies, auxillary nurse midwives, nurses, nurse midwives, clinical officers, medical assistants, doctors, students	Interprofessional	Facility-based simulation training	1 day	Basic delivery skills including active management of third stage of labour and management of PPH	Technical skills	2c	I, II	19

**Gomez et al.**	2018	Cluster-randomised controlled trial	Pretraining vs. 1–6 months posttraining vs. 7–12 months posttraining	Ghana	40 public and mission hospitals	None	Skilled birth attendants, all were registeredor certified midwives	Uniprofessional	Low-dose, high frequency training using simulators SMS quizzes and remindersMentoring	Two 4 days sessions with weekly practice sessions and support during during 1 year	Basic emergency obstetric and newborn care including newborn resuscitation, respectful maternity care and clinical decision-making	Both	1b	II, IV	8

**Grady et al.**	2011	Descriptive and pre-post study	Pretraining v.s. posttraining	Somaliland, Kenya, Malawi, Swaziland, Zimbabwe, Tanzania and Sierra Leone	Unknown	Life Saving Skills – Essential Obstetric and Newborn Care Training (LSS-EOC and NC)	Nurse-midwives, doctors, clinical officers, specialists	Interprofessional	Lectures, skills training, scenario teaching, workshops, demonstrations and discussions	3 days	Five main causes of maternal mortality, built around the nine signal functions of EOC and NC	Both	2c	I, II	12

**Hanson et al.**	2021	Cluster-randomised controlled trial	Intervention vs. control group A 6-month pretraining period vs. a 10-month posttraining period	Uganda	21 health centers, 22 hospitals	Helping Mothers Survive: Bleeding After Birth	Doctors, other medical clinicians, midwives, nurses	Interprofessional	Facility-based simulation training using a competency based methodology supported by low cost simulation materials and regular peer- supported LDHF in-situ practice Peer practice coordinators were reminded by phone calls to initiate the in facility drills	1-day, followed by drills sessions for 6–8 weeks 43 peer practice coordinators received an additional half-day training	Postpartum haemorrhage	Technical skills	1b	II, III, IV	9

**Mduma et al.**	2015	Pre-post study	Pretraining vs. posttraining	Tanzania	1 rural referral hospital	Helping Babies Breathe	All care providers working in the labor ward	Interprofessional	Hands-on practical training using a low-cost newborn simulator Followed by ‘on-the-job’ and refresher training	1 day, followed by 3-minutes weekly practice, 40-minutes monthly re-training	Basic newborn care and neonatal resuscitation	Technical skills	2c	III, IV	15

**Mduma et al.**	2018	Prospective observational study with retrospective analysis	None	Tanzania	1 rural referral hospital	Helping Babies Breathe	Maternity staff	Unknown	Hands-on practical training using a low-cost newborn simulator	1 day	Basic newborn care and neonatal resuscitation	Technical skills	5	IV	17

**Mildenberger et al.**	2017	Descriptive and pre-post study	Pretraining vs. cohort 1 12-months posttraining or vs. cohort 2 1-month posttraining	Uganda	1 public regional referral hospital, health units in the surrounding district	None	Midwives, intern doctors	Interprofessional	Workshop with a skills component	Unknown	Neonatal resuscitation	Technical skills	2c	I, II	10

**Mirkuzie et al.**	2014	Descriptive and pre-post study	Pretraining vs. post-training vs. 6 months posttraining	Ethiopia	10 public health centers	Basic Emergency Obstetrics and Neonatal Care (BEmONC)	Midwives, nurses	Interprofessional	Hands-on skills training using low-cost and low-tech simulators	18 days	Basic emergency obstetric and neonatal care topics	Technical skills	2c	I, II	16

**Msemo et al.**	2013	Pre-post study	Pretraining vs. posttraining	Tanzania	3 referral hospitals, 4 regional hospitals, 1 district hospital	Helping Babies Breathe	Health care providers, major emphasis was placed on midwives	Unknown	Hands-on practical training using a low-cost newborn simulator Followed by ‘on-the-job’ and refresher training	1 day, followed by ‘on-the-job’ and refresher training	Basic newborn care and neonatal resuscitation	Technical skills	2c	III, IV	9

**Nelissen**	2015	Pre-post study	Pretraining vs. posttraining vs. 9 months posttraining	Tanzania	1 rural referral hospital	Helping Mothers Survive: Bleeding After Birth	Clinicians, nurse-midwives, medical attendants, ambulance drivers, other staff involved in maternity care	Interprofessional	Facility-based simulation training	Half day	Basic delivery skills including active management of third stage of labour and management of PPH	Technical skills	2c	II	16

**Nelissen et al.**	2017	Pre-post study	Pretraining vs. posttraining	Tanzania	1 rural referral hospital	Helping Mothers Survive: Bleeding After Birth	Clinicians, nurse-midwives, medical attendants, ambulance drivers	Interprofessional	Mix of theory and hands-on obstetric simulation-based training using a low-cost low-tech simulator	Half day	Basic delivery skills including active management of third stage of labour and management of PPH	Technical skills	2c	III, IV	10

**Pattinson et al.**	2018	Pre-post study	Pretraining vs. posttraining	South Africa	51 community health centres, 62 district hospitals	Essential Steps in Managing Obstetric Emergencies and Essential Obstetric Training programme (ESMOE-EOST)	Healthcare professionals involved in maternity care	Interprofessional	Off-site skills and drills training	3 days for professionals from district hospitals 2 days for professionals from community health centres	Direct causes of maternal death, labour care, neonatal resuscitation, and prevention of transmission of HIV	Technical skills	2c	II, IV	9

**Pattinson et al.**	2019	Pre-post study	Pretraining vs. posttraining Intervention vs. control group	South Africa	12 healthcare districts (intervention group), 40 healthcare districts (comparison group)	Essential Steps in Managing Obstetric Emergencies and Essential Obstetric Training programme (ESMOE-EOST)	Doctors, midwives, nurses, others	Interprofessional	Off-site skills and drills training	Junior midwives 2 days, senior midwives/all medical staff 3 days Monthly ‘fire drills’	Major causes of maternal and newborn death, including EmOC signal functiond and recognition and management of complications in HIV positive women	Technical skills	2c	IV	13

**Reynolds et al.**	2017	Descriptive study	None	Guinea-Bissau	Regional hospitals and different types of health units	CONU (Cuidados Obstétricos e Neonatais de Urgência) training programme	Nurses, midwives, doctors	Interprofessional	Interactive and practical sessions, using demonstrative and simulation-based training	60 hours (15 sessions of 4 hours) over 8 weeks	Obstetric and neonatal urgent care	Both	5	I, II	28

**Rosenberg et al.**	2020	Pre-post study	Pretraining vs. posttraining	Rwanda	Referral, provincial, district hospitals	Emergency Obstetric and Neonatal Care Course (EONC)	EONC1: nurses, anesthetistsEONC2: midwives, nurses, physicians	Interprofessional	Prehospital skills stations, simulation, didactics	2 days	Management of prolapsed umbilical cords, delivery of twins, breech delivery, shoulder dystocia, and newborn resuscitation among others	Technical skills	2c	II	13

**Rule et al.**	2017	Pre-post study	Pretraining vs. posttraining	Kenya	1 rural referral, teaching hospital	Helping Babies Breathe	All staff who took care of mothers and babies	Interprofessional	Hands-on practical training using a low-cost newborn simulator coupled withquality improvement approaches	1 day	Basic newborn care and neonatal resuscitation	Technical skills	2c	IV	19

**Sorensen et al.**	2011	Pre-post study	Pretraining vs. posttraining	Tanzania	1 regional, referral hospital	Advanced Life Support in Obstetrics (ALSO)	Mid- and high-level providers involved in childbirth	Unknown	Lectures, workshops (a quiz, an AMTSL hands-on station, a teamwork-based role play) and case discussions	2 days	Postpartum haemorrhage	Both	2c	III, IV	18

**Tuyisenge et al.**	2018	Descriptive study	None	Rwanda	8 hospitals	Continuing Professional Development (CPD) program (a part of the Maternal, Newborn and Child Health in Rwanda (MNCHR) project)	Nurses, midwives, physicians	Interprofessional	Advanced Life Support in Obstetrics® (ALSO®) module, one of the five modules in the CPD program	Unknown	Obstetrical emergencies	Technical skills	5	I	6

**Ugwa et al.**	2020	Cluster randomized controlled trial	Intervention vs. control group Pretraining vs. posttraining vs. 3 months posttraining vs. 12 months posttraining	Nigeria	60 health facilities	None	Community health extension workers, doctors, nurses, others	Interprofessional	1. Onsite simulation-based, team-oriented, LDHFT plus mobile mentoring 2. Offsite lectures with practice sessions on simulators, group-based training approach	1. 2 training courses of 4 days each, with additional time for assessment as needed with brief, ongoing activities2. 8 days	Basic EmergencyObstetric and Newborn Care (BEmONC) functions	Both	1b	I, II	21

**Umar et al.**	2018	Descriptive and pre-post study	Pretraining vs. posttraining	Nigeria	34 general hospitals, 3 teaching hospitals, 1 federal medical center, 2 specialist hospitals, 4 comprehensive health centers	None	Doctors, midwives, nurses	Interprofessional	Lectures, skills and scenario demonstrations using simulators	1 day	Neonatal resuscitation	Technical skills	2c	II	10

**Van Tetering et al.**	2021	Descriptive and pre-post study	Pretraining vs. posttraining	Uganda	1 national referral hospital	Training for life	Residents	Uniprofessional	A technology-enhanced simulation-based training focusing on medical technical skills and teamwork	1 day with at least onehalf-day repetition training session	Acute obstetric scenarios focusing on medical technical skills and teamwork/crew resource management	Both	2c	I, II	20

**Walker et al.**	2020	Cluster randomized controlled trial	Intervention vs. control group	Kenya, Uganda	Kenya: 14 public, 2 non-profit missionary facilitiesUganda: 2 public and 2 non-profit missionary facilities	East Africa Preterm Birth Initiative (PTBi-EA)	Trainees: maternity ward and newborn care providers, quality improvement team membersMentors: nurses in Kenya, nurses and physicians in Uganda	Interprofessional	Intervention group: additionally to the control group quality improvement collaboratives and an adapted PRONTO International obstetric and newborn simulation and team training curriculum modified for preterm birth Control group: maternity register data strengthening, use of a locally modified WHO Safe Childbirth Checklist to enhance preterm birth identification and management	Quality improvement collaboratives: 5 learning sessionsPRONTO activities: 58h	Intrapartum and immediate newborn package with a focus on preterm birth	Both	1b	IV	9

**Willcox et al.**	2017	Cost-effectiveness study	The cost and incremental cost-effectiveness of training vs. no training	Ghana	40 regions, public and mission hospitals	None	Midwives, nurses	Interprofessional	Low-dose, high-frequency onsite simulation-based training, mentorship and coaching	Two 4 days sessions	Basic obstetric care, followed by training in emergency maternal and newborn care	Both	2b	IV	15

**Williams et al.**	2019	Descriptive study	Three training approaches	Uganda	125 facilities including health centers level III, IV and hospitals Qualitative data came from 24 selected facilities	None (based on Helping Babies Breathe and Helping Mothers Survive: Bleeding After Birth)	All maternity unit staff	Interprofessional	1. Facility-based, LDHF team training and ongoing practice2. As group 1 + peer-assisted learning component3. As group 2 + phone support	1 day HMS BAB with suggestion to practice for 10–15min once per week for 8 weeks, followed by 1 day HBB training, with suggestion to practice 10–15min once per week for 8 weeks, followed by suggestion to practice both maternal and newborn scenarios for 4 weeks	Postpartum haemorrhage and neonatal resuscitation	Both	5	I, III	14

**Yigzaw et al.**	2019	Quasi-experimental trial	Intervention vs. control group Pretraining vs. 3 months posttraining	Ethiopia	Health centers in 3 major regional states	None	Midwives, nurses, health officers	Interprofessional	1. Blended learning: offsite training followed by SMS and phone calls2. Conventional learning: offsite training followed by a facility visit to mentor participants	1. 12 days, followed by daily SMS and weekly phone calls2. 18 days, followed by a facility visit to mentor participants	Basic EmergencyObstetric and Newborn Care (BEmONC) signal functions	Technical skills	2b	II, IV	12

**Zanardo et al.**	2010	Descriptive and pre-post study	Pretraining vs. posttraining	Democratic Republic of Congo	Unknown	Neonatal Resuscitation Course and workshop on Laryngeal Mask Airway	Physicians, midwives	Interprofessional	Didactic sessions, followed by practical, hands-on workshop with a neonatal manikin	3 days	Neonatal resuscitation program including laryngeal mask airway positioning and bag-ventilation	Technical skills	2c	I, II	12

**Zongo et al.**	2015	Cluster-randomised controlled trial	Caesarean section vs. vaginal delivery	Mali, Senegal	22 health care facilities in Mali, 24 health care facilities in Senegal	Quality of care, Risk management and Technology in obstetrics (QUARITE)	Doctors, midwives, nurses	Interprofessional	Interactive workshop using the ALARM international course and outreach visits focused on maternal death reviews and best practice implementation	6-days workshop, quarterly educational outreach visits during2 years	Emergency obstetric care, topics were based on maternal death reviews. Most recurrent topics were pre-eclampsia and management of PPH	Both	1b	IV	11


Thirteen of the 47 included articles were related to the Helping Babies Breath program [22,25,72,73,74,27,42,49,51,65,69,70,71], and eight to the Helping Mothers Survive: Bleeding After Birth program [34,42,43,49,75,76,77,78]. Over the years, the insights gained from evaluations of these training programs have led to the modification and refinement of instructional design features. The addition of refresher courses to the original course program, leading to a change in the instructional design feature of repetitive practice, is an example of this. Additionally, simulation-based training programs were increasingly accompanied by other quality improvement collaboratives such as maternal death reviews, supportive supervision visits, mobile mentoring (by phone or SMS), or peer-assistant learning [26,39,52,66,79,40,41,42,43,45,47,49,51]. Most studies were conducted in Tanzania [22,28,71,74,75,78,80,30,34,51,63,64,65,69,70], Ghana [27,33,41,47,54,60,80,81], Kenya [45,58,63,72,73,80], Uganda [42,43,45,49,57,61], and Malawi [36,63,64,80]. The range of involved hospitals spans the whole spectrum from rural health clinics to tertiary teaching hospitals.

### Study population and duration

Participants of the training programs included providers from all healthcare levels in paediatrics, obstetrics, anaesthetics, and ambulance drivers. In six studies training was set up uniprofessional [25,27,35,41,54,61] and in 37 studies interprofessional [31,32,45,47,48,49,51,52,55,56,58,60,33,62,63,64,65,70,71,72,73,74,75,34,77,78,81,82,83,84,85,36,37,38,40,43,44]. Twenty-seven studies concentrated on technical skills [21,22,48,51,52,54,56,62,64,65,69,71,25,72,73,74,78,84,85,86,27,32,34,37,38,42,43], one study on non-technical skills [35], and nineteen on both technical and non-technical skills [30,33,63,68,79,80,81,82,83,87,88,36,45,47,49,55,58,60,61]. The total duration of the training exhibited a notable variability, spanning from a half day to a 18-day training. The diversity in training duration was complemented by a broad spectrum of repetitive training schedules, encompassing intervals ranging from annual repetitions to weekly sessions over the span of a year. The duration of the repetition training also varied between three minutes up to a half day of training. As the years have progressed, an increasing inclusion of repetitive training schedules has been observed.

### Outcome measures on Kirkpatrick’s four levels

Table 1 gives an overview of all evaluated levels of Kirkpatrick’s model. Eighteen studies described results on Kirkpatrick level 1 [36,44,61,62,63,64,65,75,83,84,47,48,49,54,55,56,58,60]. All studies showed positive reactions, and challenges and recommendations were faced in twelve studies (Table 2). These challenges include frequent staff rotation, work schedules that prevented trainees from attending training, and low financial incentives [48,49,65,79]. The recommendation to extend training duration and adding refresher training sessions was made in nine articles [48,50,55,57,58,60,61,63,65].

**Table 2:** Main findings of the included studies categorized by Kirkpatrick levels.


AUTHORS	YEAR	OUTCOME MEASURES (KIRKPATRICK LEVEL)	KIRKPATRICK LEVEL I: REACTION	KIRKPATRICK LEVEL II: LEARNING *INCLUDING INDEPENDENT PREDICTORS OF TRAINING RESULTS*	KIRKPATRICK LEVEL III: BEHAVIOR	KIRKPATRICK LEVEL IV: RESULTS	OTHER RESULTS

**Afulani et al.**	2019	IV	–	–	–	An increase in person-centered maternity care scores Subscales dignity and respect, communication and autonomy, and supportive care increased	–

**Afulani et al.**	2020	I, II	Participants agreed that the training was useful, that they will use the tools, that they noted improvements in their knowledge and confidence, as well in patient-provider communication and teamwork *Recommendations:* increasing the length of the training, adding more sessions, and holding the training more frequently. A suggestion of shorter days of training over a longer period was made. Other recommendations include to see more providers and medical staff, to cover more clinical and respectful maternity care topics, to tailor the simulations to the different levels of facilities. Concerns regarding its financial sustainability.	Improvement in knowledge and self-efficacy	–	–	–

**Al-Beity et al.**	2020	II	–	Improvement in knowledge and skills across all professions Retention at 10-months follow-up was high *Independent predictors for better skill outcomes and less decline 10 months posttraining:* profession and number of deliveries in the last month	–	–	–

**Ameh et al.**	2012	I, II, III	Enjoyment of the training and participants reported that the skills and knowledge acquired would be useful in performing their jobs better *Recommendations:* to include sessions on record keeping and quality of care, to increase the duration of training from 4 to 7 days, to enable more practice on mannequins, shortage of equipment and drugs limite to perform some of the skills taught	Improvement in knowledge and skills	An increase in confidence in responding to obstetric emergencies in a structured and logical way The labor ward was reorganized after the training	An increase in the number of available signal functions All 3 hospitals were able to provide all emergency obstetric signal functions following the training Midwives provided additional signal functions that had previously been provided only by medical doctors	Some midwives reported that they were not able to perform some signal functions, because of the hospital policy

**Ameh et al.**	2016	II	–	Improvement in knowledge and skills among all cadres and countries Independent predictors of a higher pretraining score: a teaching job, previous in-service training, higher percentage of work time spent providing maternity care	–	–	Those with more than 11 years of experience in obstetrics had the lowest scores prior to the training, with mean improvement ratios 1.4% lower than for those with no more than 2 years of experience

**Andreatta et al.**	2011	I, II, III, VI	Training was valuable and effective for acquiring and maintaining skills	Improvement in skills	13 incidences of PPH were controlled using bimanual uterine compression	No maternal mortality after training	Skills performances were different per cadre

**Arabi et al.**	2016	II	–	Improvement in skills 3 and 12 months post-training Assessments 3 and 12 months post-training showed low scorings on the skill ‘preparation for birth’ section mainly due to failure to demonstrate the subitem of ‘clean hands’ At 12-monhs stimulation of the non-breathing manikin almost doubled	–	–	–

**Arlington et al.**	2017	I, II	High satisfaction levels Feeling more confident and more skilled *Recommendations:* training was too short, financial incentives were too small, intrafacility rotation of trained attendants limited the impact of the training. The supportive visits and follow-up visits are critical for skill retention	Structured on-the job training and supportive supervisory visits were associated with improvement in skill retention A reduction in skills after 4–6 weeks and 4–6 months Independent predictors of passing the skills test were: time since training, facility level, and health cadre	–	–	–

**Asiedu et al.**	2019	I	Participants felt that the training strengthened in their technical capacity and confidence, facilitated translation of skills into routine service delivery, and improved the quality of the maternal and newborn services they provided *Challenges and recommendations* have also been noted	–	–	–	–

**Bang et al.**	2016	II	–	Improvement in knowledge and skills Skills decreased more than knowledge over time Independent predictors of deterioration of Objective Structured Clinical Examinations skills (OSCE): facility type and prior training	–	–	–

**Cavicchiolo et al.**	2018	II	–	Most non-technical skills were scored as poor or marginal Small improvements were observed in task management after the first course. Limited improvements were observed in task management and decision-making after the low-dose/high-frequency training. No differences were observed in situation awareness, apart from a small improvement in recognizing/understanding.	–	–	–

**Chang et al.**	2019	I, III, IV	An improvement in hospital safety culture scores	–	An increase in the use of postpartum hemorrhage procedural interventions	A decrease in the rate of maternal mortality from obstetric hemorrhage No change in the rate of obstetric hemorrhage, uterine atony, and hysterectomy	–

**Chaudhury et al.**	2016	IV	–	–	–	Cost per trainee $151, cost per health facility $602, and cost per facility for each re-training $173 The estimated total for all Tanzania initial rollout lies between $2 934 793 to $4 309 595. It would cost $ 2 019 115 for a further one year and $ 5 640 794 for a further five years of ongoing program support	–

**Dettinger et al.**	2018	I, II, III	The PRONTO intervention was extremely useful Enjoyment of the simulation and teamwork components and participants would like to implement teamwork and the practiced skills *Recommendations*: to extend duration-frequency of the training, to change the training space to a larger venue, to expand clinical content	Improvement in knowledge, self-efficacy, and self-reported teamwork Improvement retained after 3 months	A high proportion of facilities achieving self-defined strategic goals	–	–

**Drake et al.**	2019	II	–	Similar average skills scores between initial and modified training groups immediately post-training. Both groups experienced drops in skills over time. The modified training approach was associated with higher skills scores 4–6 weeks post training versus the initial training approach. Medical attendant cadre showed the greatest skills retention	–	–	–

**Dumont et al.**	2013	IV	–	–	–	A higher decrease in maternal mortality in intervention hospitals than in control hospitals This effect was limited to capital and district hospitals	–

**Eblovi et al.**	2017	II, IV	–	A decrease in skills from immediately post-training to 4 months later 4 months after refresher course, skills improved to the same high level attained initially	–	5% of neonates required bag-mask ventilation 0.71% of neonates did not survive, compared with a nationwide first 24-hour mortality estimate of 1.7%.	–

**Egenberg et al.**	2017	I	Enhancement of self-efficacy and reduction in perception of stress. Perception of improved teamwork approach and skills *Recommendations:* training to be continued and disseminated, the importance of team training as learning feature	–	–	–	–

**Ersdal et al.**	2013	II, III	–	Improvement in skills	No transfer to clinical practice, no change in the number of babies being suctioned and/or ventilated at birth A decrease in the use of stimulation in the delivery room An increase in the mean time from birth to initiation of face mask ventilation	–	High confidence was related to reduced performance The number of providers who reported themselves as ‘always confident’ decreased after training

**Evans et al.**	2014	I, II	Training methods, materials and time were highly acceptable among all cadres and countries Ratings were highest for having enoughtrainers, use of the simulator as a teaching tool, and training with different provider types combined	Improvement in knowledge and self-reported confidence among all cadres and countries The largest increase and passing rate was among auxiliary nurse midwives	–	–	–

**Evans et al.**	2018	II, III, IV	–	Simulated skills maintenance for PPH prophylaxis remained high across the control, partial, and full training group 7 to 8 months after the intervention Simulated skills for newborn bag-and-mask ventilation remained high only in the full training group	An increase in uterotonic coverage within one minute in all groups Improvements in uterotonic coverage remained higher across all groups 6 months after the intervention Observed care of mother and newborn improved in all groups	A decrease in incidence of PPH and retained placenta for all groups combined A decrease in fresh stillbirths and newborn deaths for all groups combined. This remained reduced 6–9 months post-implementation. No differences were found between the three training groups.	–

**Gomez et al.**	2018	II, IV	–	Improvement in knowledge and skills Most retained after 1 year	–	A decrease in 24-hour newborn mortality after 1–6 months and 7–12 months A decrease in intrapartum stillbirths after 1–6 months and 7–12 months	Regional-level facilities had a greater risk of 24-h newborn mortality compared to district-level facilities and polyclinics No difference in the mortality rates was found when a master mentor was present

**Grady et al.**	2011	I, II	Participants expressed a high level of satisfaction with the training. The training package was found to meet the needs of healthcare providers, increased awareness of the need for evidence-based care and encouraged teamwork *Challenges*: poor quality of the meals provided, insufficient money provided by the sponsor to meet the costs of attending, length of course too short, delivery of lectures too fast with insufficient pictures and teaching equipment not working well	Improvement in knowledge and skills	–	–	–

**Hanson et al.**	2021	II, III, IV	–	Improvement in knowledge and skills	A decrease in the number of women with PPH who received oxytocin for treatment of PPH	A reduction of PPH near misses in the intervention compared to the comparison districts An increase in overall reported near miss cases and an increase in PPH case fatality rate	–

**Mduma et al.**	2015	III, IV	–	–	An increase in the number of neonates being stimulated and suctioned A decrease in neonates receiving bag mask ventilation An increase in prepartion of the resuscitation kit before delivery An increase in responsibility taken by the midwives in conducting resuscitations	A decrease in neonatal mortality at 24-h	–

**Mduma et al.**	2018	IV	–	–	–	An improvement in perinatal survival Some variations throughout the study period could be linked to different interventions and events	–

**Mildenberger et al.**	2017	I, II	Participants were very satisfied *Recommendations*: lack of refresher training	Improvement in skills and knowledge Post-testing revealed a slight decrease in skills and knowledge scores over 1 month (Cohort 2) and a significant decrease in scores over 12 months (Cohort 1)	–	–	

**Mirkuzie et al.**	2014	I, II	Training was rated appropriate and updated knowledge and skills *Recommendations:* training facilities and arrangements were unsatisfactory	An independent predictor for recording knowledge-based mastery 6 months post-training was: profession Female participants were over 3 times more likely to fail the post-course knowledge assessment compared to their male counterparts The mean immediate post-training knowledge score was 83.5% and 40% did not achieve knowledge-based mastery in their first attempt. Mean knowledge score 6 months posttraining was 80.2% and 40% have scored knowledge-based mastery (knowledge scores sustained 6 months)	–	–	–

**Msemo et al.**	2013	III, IV	–	–	An increase in the use of stimulation and suctioning A decrease in the use of face mask ventilation	A reduction in early neonatal deaths in the first 24 hours A reduction of fresh stillbirths A reduction of early perinatal mortality	–

**Nelissen et al.**	2015	II	–	Improvement in knowledge, skills and confidence Knowledge decreased after 9 months close to pre-training level Simulated basic delivery skills decreased after 9 months, simulated obstetric emergency skills were largely retained after 9 months Confidence largely retained after 9 months	–	–	–

**Nelissen et al.**	2017	III, IV	–	–	An increase in the proportion of women that received appropriate management of AMTSL and PPH	A decrease in the incidence of PPH	–

**Pattinson et al.**	2018	II, IV	–	Improvement in knowledge and skills	–	Modest improvements in the ability of community health centres and district hospitals to perform basic and comprehensive emergency obstetric and neonatal care, with regard to the number of signal functions	–

**Pattinson et al.**	2019	IV	–	–	–	A reduction in the number of maternal deaths and in the number of maternal deaths from direct and indirect obstetric causes A greater reduction in all categories of causes of maternal death in the intervention districts than in the comparison districts	–

**Reynolds et al.**	2017	I, II	Most participants rated the pedagogical variables as good or very good	Knowledge was higher among participants with 2 to 9 years of practice as compared to those with 1 year or less, or 10 or more years of practice	–	–	–

**Rosenberg et al.**	2020	II	–	An increase in knowledge of both EONC1 and EONC2 EONC1 showed improvements in knowledge, application, and problem solving, EONC2 did not	–	–	–

**Rule et al.**	2017	IV	–	–	–	A decrease in the suspected HIE rate, but this increased after initial decline An increase in the number of near-miss cases An increasing trend of birth asphyxia No change in deaths attributed to suspected HIE	–

**Sorensen et al.**	2011	III, IV	–	–	An improvement in AMTSL and management of PPH A decrease in episiotomies By visual estimation, an increase of staff identifying PPH cases	A decrease of the incidence of PPH	–

**Tuyisenge et al.**	2018	I	Participants indicated that the training had increased their knowledge and approach to maternal health care provision *Challenges*: limited opportunities to share learned knowledge among colleagues, frequent staff rotation in hospital services, the lack of refresher training and mentorship, and staff turnover	–	–	–	–

**Ugwa et al.**	2020	I, II	Participants mentioned that LDHF/m-mentoring training approach enabled to gain improvements in skills, knowledge and quality of care The respondents reported reduction in maternal and neonatal morbidity and mortality as common theme Facilitators of LDHF/m-Mentoring approach were identified as supportive *Challenges*: different work schedules prevented some trainees from attending training and unavailability of equipment hindered some from translating what they learnt into practice	Equally high mean knowledge scores between the two groups at 3 and 12 months post-training Improvements in clinical skills in both groups The observed improvement and retention of skills was higher in the intervention group compared to the control group at 12 monthspost-training	–	–	–

**Umar et al.**	2018	II	–	Variable improvements of knowledge Residents obtained higher pre- and post-training marks, with lower mean difference, than senior doctors and medical officers Junior nurses obtained higher pre-training scores compared to the senior nursing cadre, while the intermediate nursing cadre obtained higher post-training scores compared to senior nurses	–	–	–

**Van Tetering et al.**	2021	I, II	Most instructional design features were scored high, although intervals were large The highest mean score was given on the feature *feedback* and the lowest scores on *repetitive practice* and *controlledenvironment* The overall score for the training day was high *Recommendations:* to incorporate other members of the team, to add other scenarios, to have repetition training more often, to plan more time for the debriefing, especially relating to a real-life setting, and to provide the training materials a day earlier	Improvement in knowledge No changes in teamwork and (most) medical technical skills	–	–	–

**Walker et al.**	2020	IV	–	–	–	A reduction in fresh stillbirth and neonatal death (combined) among preterm and low-birthweight infants Also a reduction in perinatal mortality (fresh stillbirth and 7-day mortality), pre-discharge newborn mortality, preterm fresh stillbirth, preterm neonatal mortality	–

**Willcox et al.**	2017	IV	–	–	–	Based on previous results, 544 lives were saved during the follow-up period of 1 year. This can be translated to $1497,77 per life saved or $53,07 per DALY averted The training program as compared to no training has 100% probability of being cost-effective above a willingness to pay threshold of $1480	–

**Williams et al.**	2019	I, III	Facilitating factors: simulators were acceptable in use, practice coordinator increases number of practise sessions, phone support motivates for practice sessions, practice sessions necessary for maintaining skills. skills *Challenges*: viewing practice as routine care, heavy volume and low staffing, lack of outside support, lack of compensation	–	Simulator-based practice sessions occurred more frequently in facilities where one or two practice coordinators helped to schedule and lead the practice sessions, and in health centers compared to hospitals	–	–

**Yigzaw et al.**	2019	II, IV	–	Knowledge scores were similar for the blended and conventional learning groups before training and three months post-training with no difference in gains made Post-training skills scores were significantly higher for conventional thanblended learning Males outperformed females in knowledge, and providers with a university degree had significantly higher knowledge and skills scores than those with a diploma	–	Training costs were lower for blended learning than conventional learning (1032 USD vs 1648 USD per trainee) The blended learning approach was more cost-effective than the conventional approach (cost effectiveness ratio of 14 vs 20)	–

**Zanardo et al.**	2010	I, II	All participants, with the exception ofone, expressed a high degree of approval with regard to neonatal resuscitation by laryngeal mask airway (LMA) positioning and defined it a sustainable and cost-effective procedure	Improvement in knowledge The knowledge gained by the physicians related to the LMA positioning was superior than that achieved by the midwifes Skills showed a similar high efficacy between trained physicians and midwifes	–	–	–

**Zongo et al.**	2015	IV	–	–	–	The risk of maternal mortality was lower in the intervention group among women with cesarean delivery. The intervention had no significant effect among women with vaginal delivery This differential effect was particularly marked for district hospitals and for hospital in the capital	–


Twenty-nine studies documented results at Kirkpatrick level 2 [25,27,51,52,54,55,56,58,60,61,62,63,32,64,65,70,72,80,83,84,85,86,34,35,38,41,42,43,44]. Eighteen of these studies showed improvements in participant’s knowledge levels, as evidenced by a an increase from pre-training to post-training assessments [32,34,62,63,64,72,77,80,84,85,38,41,43,44,55,58,60,61]. Moreover, fifteen studies reported on positive advancements in participants’skills [25,32,63,72,77,80,89,34,35,41,43,44,54,55,57]. Sustained improvements in knowledge and/or skills over a period of 3 to 12 months post-training were mentioned in eight studies [25,34,41,42,44,52,56,58]. A decrease in knowledge and/or skills over time was showed in six studies [27,51,65,72,77,84]. Several independent predictors of training results on Kirkpatrick level 2 were revealed, such as trainees profession, experience in obstetrics, gender, and previous training sessions (Table 2).

Twelve studies investigated the effectiveness of training at Kirkpatrick level 3 [22,29,70,90,30,36,42,43,49,54,55,58]. Seven studies described improvements of skills on the job [22,30,36,42,55,71,78], and two studies reported on organizational changes in workplace [55,58]. One study reported no transfer of skills into clinical practice [70].

Twenty-two studies evaluated outcome measures at Kirkpatrick level 4 [22,27,43,45,52,54,69,71,73,74,78,81,30,82,91,32,33,36,37,40,41,42], with eight studies describing improvements of neonatal or perinatal morbidity or mortality [22,27,41,42,45,69,71,73]. One of these studies showed that initial improvements declined over time [73]. Additionally, eight studies revealed results of improvements on maternal outcomes, mostly related to postpartum haemorrhage and maternal mortality [30,36,37,40,42,43,78,82]. Another study highlighted an increase in respectful maternity care [33]. Furthermore, two studies mentioned an improvement in signal functions (the major interventions for averting maternal and neonatal mortalities) [32,55], and three studies provided cost-estimations for training rollout [52,74,81].

Thirteen studies reported on results not only at Kirkpatrick level 4, but also at level 2 and/or 3, hence reporting on the translation of acquired skills and knowledge into on-the-job behaviours and patient outcomes [22,27,55,71,78,30,32,36,41,42,43,52,54]. Two of the included studies provided data for all four levels of Kirkpatrick’s training evaluation model [54,55].

### Instructional design features

Analysing the reported items of the 42-item ID-SIM questionnaire across the included articles, a range emerges, spanning from 6 to 28 described items per article (14.3–66.7 percent) (Table 1). Ten articles described less than 10 items [22,32,41,43,45,48,55,65,70,80], 34 articles mentioned between 10 and 20 items [25,27,42,47,49,52,54,56,58,60,61,62,30,63,64,69,71,72,73,74,75,78,81,33,82,84,85,86,34,35,36,37,38,40], and only three article stated more than 20 items [51,79,83]. The items related to the instructional design features ‘*learning strategies*’ and ‘*defined outcomes*’ emerged as the most frequently described items across the articles (Appendix 2). The items about ‘*difficulty range*’ and ‘*individualized learning*’ were rarely mentioned.

## Discussion

### Main findings

This review gives an overview of 47 studies on emergency obstetric, postgraduate, simulation-based training in sub-Saharan and Central Africa, and examines the applied instructional design features of training programs. Results comprise rising evidence that training can have a positive impact across all four levels of Kirkpatrick’s training evaluation model. However, results were not consistent across all studies and the effects vary over time. To understand why some simulation-based training programs were more effective than others, we incorporated a quality assessment of the instructional design within the evaluated training programs. However, the heterogeneous nature of descriptions for instructional design items introduced a significant challenge to achieve an objective scoring. In fact, the number of described instructional design items varied between 14.3 and 66.7 percent, with only three out of 47 articles describing more than 20 out of 42 items.

In general, the results of this review on Kirkpatrick’s levels of training evaluation correspond with the findings of other reviews that evaluate emergency obstetric simulation-based training including other geographical regions than sub-Saharan and Central Africa. One literature review about emergency obstetric and neonatal care training in high-income and low- and middle-income countries focused on Kirkpatrick levels 3 and 4, and reported mostly positive changes in behaviour, the process, and patient outcomes [92]. A subsequent review about the effectiveness of training in emergency obstetric care in high-income and low- and middle-income countries noted improvements in healthcare providers knowledge, skills, clinical practice, and neonatal outcomes [93]. However, the strength of evidence for a reduction in stillbirths, maternal morbidity, and maternal mortality was less strong [93]. Another review by Brogaard et al. about obstetric emergency team training in high-resource settings suggests a positive effect on some neonatal outcomes, but also stated conflicting results on neonatal and maternal outcomes [94]. Finally, Fransen et al. assessed the effects of simulation-based obstetric team training in high-income and low- and middle-income countries, and included only randomised controlled trials in their review [8]. Results of eight included studies showed that training, compared with no training, may help to improve team performance of obstetric teams, and that it might contribute to improvement of specific maternal and perinatal outcomes [8]. Both Brogaard et al. and Fransen et al. highlighted the need to undertake future high-quality studies, including comparisons between training courses with a different instructional design, to identify the optimal methodology for effective team training [8,94].

The majority of included studies in this review reported positive results when evaluating their training program on patient outcomes. This effect may be partly due to the higher incidence of adverse maternal and perinatal outcome in sub-Saharan and Central Africa, allowing for an easier detection of a change. The high prevalence of positive training results could also potentially be influenced by publication bias favouring positive outcomes. The observed lower emphasis on the instructional design of training programs in sub-Saharan and Central Africa can be attributed to a combination of factors such as unfamiliarity of instructional design items, and resource limitations prevalent in these regions, including inadequate staffing and constrained budgets. The staff may prioritize clinical work and providing training, instead of evaluating and improving training programs.

An aspect to bear in mind is the original intention of the ID-SIM questionnaire, which was designed to assess instructional design features within the context of simulation-based team training. However, the scope of this review encompassed training programs that targeted uniprofessional training as well. Some of the instructional design items may be less relevant for uniprofessional training programs, what may have resulted in bias in the number of described items. An additional layer of complexity arises from the practice observed in some articles, wherein reference is made to prior publications that delve into the same training program. As we based the scoring on the information provided in the current article only, this may have led to underreported items. Combing the results of the articles on the same training programs (Helping Babies Breath project (23–32), Helping Mothers Survive: Bleeding After Birth program (33–36), QUARITE study (37,38)) did not give an objective result, because evaluation of these training programs resulted in modification of instructional design features over the years. Hence, the evaluation of the instructional design of training programs with a single name, may still differ per location and moment.

### Strengths and limitations

The strength of this review is that we did not solely overview studies on emergency obstetric, postgraduate, simulation-based training in sub-Saharan and Central Africa, but also examined the applied instructional design of training programs. Two authors independently assessed all published studies and selected the studies for inclusion in order to minimize bias. Four authors performed the data extraction, data synthesis, and quality of evidence assessment. Any disagreements were resolved by discussion between the authors or, if required, by consultation of another author. Analyses was performed with a narrative syntheses, rather than meta-analyses, as studies were heterogenous with regard to design, training program, and measures of effectiveness. Most included studies in this review used pre-post study designs. While these designs offer valuable insights into training impact, they also introduce potential bias arising from concurrent events or changes that might have occurred during the training evaluation periods. An essential aspect to bear in mind is the challenge posed by the heterogeneous descriptions of instructional design items across the reviewed studies. As a consequence, it was impossible to explore a potential correlation between ID-SIM scores and the effectiveness of training programs.

### Implications for practice

The rationale for focusing on sub-Saharan and Central Africa was due to the persisting high number of deaths due to complications related to pregnancy and childbirth [1]. Challenges in these areas comprise the wide variation in local settings including under-resourced health services, inadequate medical staff, and regular rotation of medical staff. Under these circumstances, perhaps with the most need for training, appropriate knowledge of simulation-based training in obstetrics will be useful to develop and evaluate sustainable, clinically effective training programs [95]. This review showed that additional evidence is available that emergency obstetric simulation-based training can have a positive impact in sub-Saharan and Central Africa, but also that future high-quality studies are necessary to identify the optimal methodology for most effective training. Over the years, simulation-based training programs were increasingly accompanied by other quality improvement collaboratives such as maternal death reviews and supportive supervision visits. In the context of sub-Saharan and Central Africa, the choice to opt for on-site training over off-site venues may create the opportunity to reach more staff members by avoiding the logistical challenges of going to a simulation center. Another advantage of on-site training is that it generates more suggestions for organizational changes compared to off-site simulation training [96]. Another implication for practice is to include non-technical skills during emergency obstetric simulation-based training in sub-Saharan and Central Africa. While most studies in this review mainly focused on technical skills, training of non-technical skills became more frequently part of training programs. Development of non-technical skills such as situational awareness, decision-making, communication, teamwork, and leadership may be even more important while managing emergency obstetric and neonatal conditions in the complex healthcare landscape of sub-Saharan and Central Africa.

### Recommendations for future research

To attain a comprehensive understanding of the mechanisms that determines why certain training programs are more effective in improving maternal and neonatal healthcare outcomes than other, the imperative lies in conducting robust, well-designed studies including detailed descriptions of instructional design features of the evaluated training programs. Most included studies in this review were pre-post design studies. Nevertheless, the design of the studies became stronger over the years through including control groups and setting up randomized controlled trials.

## Conclusion

This review provides an overview of 47 articles about emergency obstetric, postgraduate, simulation-based training in sub-Saharan and Central Africa. Results of these studies comprise rising evidence that training can have a positive impact across all four levels of Kirkpatrick’s training evaluation model. However, results were not consistent across all studies, and the effects vary over time. To understand why some simulation-based training programs were more effective than others, we incorporated a quality assessment of the instructional design within the evaluated training programs. However, instructional design items were heterogeneously applied and described, what made objective scoring and comparing of the items impossible. A detailed description of the instructional design features of a training program will contribute to a deeper understanding of the underlying mechanisms that determine why certain training programs are more effective in improving maternal and neonatal healthcare outcomes than others.

## Additional File

The additional file for this article can be found as follows:

10.5334/aogh.3891.s1 Appendices. Appendix 1 and 2.
