# Supplementary material for: Simulation-Based Training in Emergency Obstetric Care in Sub-Saharan and Central Africa: A Scoping Review
Source: Ann Glob Health. 2023 Sep 28;89(1):62. doi: 10.5334/aogh.3891 (PMC10540704; doi:10.5334/aogh.3891)
Supplement: Appendices. — Appendix 1 and 2. [file agh-89-1-3891-s1.pdf]

Appendix

Appendix 1 Search

Search Pubmed

Obstetrics[Mesh] OR Obstet*[tiab] OR Delivery, Obstetric[Mesh] OR Deliver*[tiab] OR Labor, Obstetric [Mesh] OR labor[tiab] OR labor[tiab] OR Midwifery [Mesh] OR midwife*[tiab] OR midwiv*[tiab] OR Traditional Birth Attendant*[tiab]

Simulation Training[Mesh] OR ((simulation*[tiab] OR interactive*[tiab] OR inter active*[tiab] OR Manikins[Mesh] OR Manikin*[tiab] OR Mannequin*[tiab] OR in-situ[tiab] OR Inservice Training[Mesh] OR in service[tiab] OR inservice[tiab]) AND (training*[tiab] OR educat*[tiab] OR learning*[tiab]))

Africa South of the Sahara[Mesh] OR sub saharan Africa[tiab] OR Central Africa[tiab] OR Cameroon*[tiab] OR Central African Republic[tiab] OR Ubangi-Shari[tiab] OR Chad[tiab] OR Congo*[tiab] OR Kinshasa*[tiab] OR Zaire*[tiab] OR Katanga*[tiab] OR Guinea*[tiab] OR rio Muni[tiab] OR Gabon*[tiab] OR Sao Tome and Principe*[tiab] OR Eastern Africa*[tiab] East Africa*[tiab] OR British Indian Ocean Territor*[tiab] OR Burundi*[tiab] OR Urundi*[tiab] OR Djibouti*[tiab] French Somaliland*[tiab] OR Eritrea*[tiab] OR Ethiopia*[tiab] OR Keny*[tiab] OR Kenia*[tiab] OR Rwanda*[tiab] OR Ruanda*[tiab] OR Somalia*[tiab] OR Sudan*[tiab] OR Tanzania*[tiab] OR Zanzibar*[tiab] OR Tanganyika*[tiab] OR Uganda*[tiab] OR Southern Africa*[tiab] OR Angola*[tiab] OR Botswana*[tiab] OR Bechuanaland*[tiab] OR Kalahari*[tiab] OR Lesotho*[tiab] OR Basutoland*[tiab] OR Malawi*[tiab] OR Nyasaland*[tiab] OR Mozambique*[tiab] OR East Africa*[tiab] OR Namibia*[tiab] OR South Africa*[tiab] OR Swaziland*[tiab] OR Zambia*[tiab] OR Rhodesia*[tiab] OR Zimbabwe*[tiab] OR Western Africa*[tiab] OR Benin*[tiab] OR Dahomey*[tiab] OR Burkina Faso*[tiab] OR Upper Volta*[tiab] OR Cabo Verde*[tiab] OR Cote d'Ivoire*[tiab] OR Ivory* OR Gambia*[tiab] OR Ghana*[tiab] OR Gold Coast*[tiab] OR Guinea*[tiab] OR Liberia*[tiab] OR Mali*[tiab] OR Mauritani*[tiab] OR Niger*[tiab] OR Senegal*[tiab] OR Sierra Leone*[tiab] OR Togo*[tiab]

Search Embase

1 exp obstetrics/ (34233)
2 exp obstetric delivery/ (126964)
3 exp labor/ (32520)
4 exp midwife/ (28498)
5 (Deliver* or labor or labor or midwife* or midwiv* or Traditional Birth Attendant*).ab,kw,ti. (858653)
6 exp "Africa south of the Sahara"/ (211041)
7 (((Africa South of the Sahara or subsaharan Africa or Central Africa* or Cameroon* or Ubangi-Shari or Chad or Congo* or Kinshasa* or Zaire* or Katanga* or Guinea* or rio Muni or Gabon* or Sao Tome) and Principe*) or Eastern Africa* or East Africa* or British Indian Ocean Territor* or Burundi* or Urundi* or Djibouti* French Somaliland* or Eritrea* or Ethiopia* or Keny* or Kenia* or Rwanda* or Ruanda* or Somalia* or Sudan* or Tanzania* or Zanzibar* or Tanganyika* or Uganda* or Southern Africa* or Angola* or Botswana* or Bechuanaland* or Kalahari* or Lesotho* or Basutoland* or Malawi* or Nyasaland* or Mozambique* or East Africa* or Namibia* or South Africa* or Swaziland* or Zambia* or Rhodesia* or Zimbabwe* or Western Africa* or

Benin* or Dahomey* or Burkina Faso* or Upper Volta* or Cabo Verde* or Cote d'Ivoire* or Ivory* or Gambia* or Ghana* or Gold Coast* or Guinea* or Liberia* or Mali* or Mauritani* or Niger* or Senegal* or Sierra Leone* or Togo*).ab,kw,ti. (1058297)
8 6 or 7 (1108282)
9 1 or 2 or 3 or 4 or 5 (947207)
10 exp simulation training/ (2928)
11 exp manikin/ (1251)
12 exp in service training/ (14956)
13 (Simulation* or interact* or inter active* or Manikin* or Mannequin* or in-situ or in service* or inservice).ab,kw,ti. (2075116)
14 11 or 12 or 13 (2088547)
15 (training* or educat* or learning*).ab,kw,ti. (1273991)
16 14 and 15 (115635)
17 10 or 16 (116415)
18 7 and 9 and 17 (610)

Appendix 2. Instructional design features per included study

Author	Name of the training program	Feedback	Repetitive practice	Curriculum integration	Difficulty range	Learning strategies	Clinical variation	Controlled environment	Defined outcomes	Individualized learning	Simulation	Number of described items (total 42 items)
Afulani et al, 2019	None (based on PRONTO international)	3/6	1/4	4/5	0/4	2/2	3/4	1/3	4/5	0/4	2/5	20
Afulani et al, 2020	None (based on PRONTO international)	3/6	1/4	4/5	0/4	2/2	3/4	1/3	3/5	0/4	2/5	19
Al-Beity et al, 2020	Helping Mothers Survive: Bleeding After Birth	1/6	4/4	4/5	1/4	2/2	2/4	2/3	1/5	0/4	1/5	18
Ameh et al, 2012	Life Saving Skills - Emergency Obstetric and Newborn Care (LSS-EOC and NC)	1/6	1/4	2/5	0/4	1/2	1/4	0/3	1/5	0/4	0/5	7
Ameh et al, 2016	Emergency Obstetric and Newborn Care (EmOC&NC)	1/6	1/4	2/5	0/4	0/2	1/4	0/3	1/5	0/4	0/5	6

Andreatta et al, 2011	None	0/6	2/4	1/5	0/4	2/2	2/4	0/3	5/5	1/4	2/5	15
Arabi et al, 2016	Helping Babies Breathe	1/6	1/4	3/5	1/4	1/2	2/4	0/3	4/5	0/4	1/5	14
Arlington et al, 2017	Helping Babies Breathe	1/6	2/4	1/5	0/4	0/2	1/4	0/3	2/5	0/4	0/5	7
Asiedu et al, 2019	None	2/6	4/4	4/5	0/4	2/2	1/4	2/3	0/5	1/4	1/5	18
Bang et al, 2016	Helping Babies Breathe	2/6	1/4	5/5	0/4	2/2	2/4	0/3	4/5	0/4	2/5	18
Cavicchiolo et al, 2018	None	5/6	3/4	3/5	0/4	2/2	1/4	0/3	3/5	0/4	1/5	18
Chang et al, 2019	Alliance for Innovation on Maternal Health (AIM) Malawi program	1/6	1/4	4/5	0/4	2/2	3/4	0/3	4/5	0/4	0/5	15
Chaudhury et al, 2016	Helping Babies Breathe	2/6	2/4	1/5	1/4	2/2	2/4	0/3	4/5	0/4	2/5	16
Dettinger et al, 2018	PRONTO International simulation-based training	2/6	1/4	5/5	0/4	2/2	2/4	2/3	2/5	0/4	2/5	18
Drake et al, 2019	Helping Babies Breathe	1/6	3/4	5/5	1/4	2/2	2/4	1/3	5/5	0/4	1/5	21
Dumont et al, 2013	Quality of care, Risk management and Technology in obstetrics (QUARITE)	2/6	3/4	1/5	0/4	2/2	2/4	0/3	2/5	0/4	1/5	13
Eblovi et al, 2017	Helping Babies Breathe	1/6	1/4	3/5	0/4	2/2	3/4	0/3	4/5	0/4	0/5	14
Egenberg et al, 2017	Based on Helping Mothers Survive: Bleeding After Birth	2/6	1/4	3/5	0/4	2/2	2/4	0/3	1/5	0/4	1/5	12

Ersdal et al, 2013	Helping Babies Breathe	1/6	1/4	1/5	0/4	1/2	2/4	0/3	2/5	0/4	1/5	9
Evans et al, 2014	Helping Mothers Survive: Bleeding After Birth	1/6	1/4	3/5	1/4	2/2	2/4	3/3	3/5	2/4	1/5	19
Evans et al, 2018	None, used the Helping Babies Breathe and Helping Mothers Survive: Bleeding After Birth training modules	1/6	1/4	3/5	0/4	2/2	1/4	0/3	4/5	0/4	0/5	12
Gomez et al, 2018	None	0/6	1/4	1/5	0/4	2/2	1/4	0/3	2/5	0/4	1/5	8
Grady et al, 2011	Life Saving Skills – Essential Obstetric and Newborn Care Training (LSS-EOC and NC)	1/6	1/4	2/5	0/4	2/2	2/4	0/3	3/5	0/4	1/5	12
Hanson et al, 2020	Helping Mothers Survive: Bleeding After Birth	0/6	1/4	2/5	0/4	2/2	2/4	0/3	1/5	0/4	1/5	9
Mduma et al, 2015	Helping Babies Breathe	1/6	4/4	3/5	0/4	2/2	1/4	0/3	3/5	0/4	1/5	15
Mduma et al, 2018	Helping Babies Breathe	1/6	1/4	3/5	0/4	2/2	3/4	0/3	5/5	0/4	2/5	17
Mildenberger et al, 2017	None	1/6	1/4	0/5	0/4	2/2	2/4	0/3	3/5	0/4	1/5	10
Mirkuzie et al, 2014	Basic Emergency Obstetrica and Neonatal Care (BEmONC)	2/6	3/4	2/5	0/4	2/2	2/4	1/3	1/5	1/4	2/5	16
Msemo et al, 2013	Helping Babies Breathe	0/6	1/4	4/5	0/4	1/2	1/4	0/3	2/5	0/4	0/5	9
Nelissen et al, 2015	Helping Mothers Survive: Bleeding After Birth	2/6	2/4	1/5	0/4	2/2	3/4	0/3	4/5	0/4	2/5	16
Nelissen et al, 2017	Helping Mothers Survive: Bleeding After Birth	1/6	1/4	3/5	0/4	0/2	2/4	1/3	2/5	0/4	0/5	10

Pattinson et al, 2018	Essential Steps in Managing Obstetric Emergencies and Essential Obstetric Training programme	2/6	1/4	2/5	1/4	0/2	1/4	0/3	3/5	0/4	0/5	9
Pattinson et al, 2019	Essential Steps in Managing Obstetric Emergencies and Essential Obstetric Training programme (ESMOE-EOST)	0/6	2/4	4/5	0/4	2/2	1/4	1/3	3/5	0/4	0/5	13
Reynolds et al, 2017	CONU (Cuidados Obstétricos e Neonatais de Urgência) training programme	3/6	4/4	3/5	4/4	2/2	4/4	0/3	5/5	1/4	2/5	28
Rosenberg et al, 2020	Emergency Obstetric and Neonatal Care Course (EONC)	2/6	1/4	4/5	0/4	2/2	1/4	0/3	3/5	0/4	0/5	13
Rule et al, 2017	Helping Babies Breathe	1/6	2/4	4/5	0/4	2/2	2/4	1/3	5/5	0/4	2/5	19
Sorensen et al, 2011	Advanced Life Support in Obstetrics (ALSO)	1/6	3/4	1/5	1/4	1/2	1/4	1/3	3/5	1/4	2/5	18
Tuyisenge et al, 2018	Continuing Professional Development (CPD) program on Advanced Life Support in Obstetrics® (ALSO)	1/6	1/4	3/5	0/4	0/2	1/4	0/3	0/5	0/4	0/5	6
Ugwa et al, 2020	None	2/6	4/4	4/5	1/4	2/2	2/4	1/3	4/5	0/4	1/5	21
Umar et al, 2018	None	0/6	1/4	1/5	0/4	2/2	2/4	0/3	3/5	0/4	1/5	10
Van Tetering et al, 2021	Training for life	3/6	2/4	3/5	0/4	2/2	3/4	2/3	2/5	1/4	2/5	20
Walker et al, 2020	East Africa Preterm Birth Initiative (PTBi-EA)	1/6	1/4	2/5	0/4	2/2	1/4	0/3	2/5	0/4	0/5	9

Willcox et al, 2017	None	1/6	2/4	3/5	0/4	2/2	2/4	0/3	3/5	0/4	2/5	15
Williams et al, 2019	None, used the Helping Babies Breathe and Helping Mothers Survive: Bleeding After Birth training modules	1/6	1/4	3/5	0/4	2/2	2/4	2/3	1/5	1/4	1/5	14
Yigzaw et al, 2019	None	1/6	1/4	1/5	0/4	2/2	2/4	1/3	4/5	0/4	0/5	12
Zanardo et al, 2010	Neonatal Resuscitation Course and workshop	0/6	2/4	2/5	0/4	2/2	2/4	0/3	3/5	0/4	1/5	12
Zongo et al, 2015	Quality of care, Risk management and Technology in obstetrics (QUARITE)	1/6	1/4	3/5	0/4	2/2	2/4	0/3	1/5	0/4	1/5	11
**Percentage of described items per instructional design feature**		36,8	40,5	45,5	7,8	75,9	47,4	10,3	57,9	5,2	33,1	
